# Inhibition of Tissue Matrix Metalloproteinases Interferes with *Mycobacterium tuberculosis*-Induced Granuloma Formation and Reduces Bacterial Load in a Human Lung Tissue Model

**DOI:** 10.3389/fmicb.2017.02370

**Published:** 2017-12-05

**Authors:** Venkata R. Parasa, Jagadeeswara R. Muvva, Jeronimo F. Rose, Clara Braian, Susanna Brighenti, Maria Lerm

**Affiliations:** ^1^Division of Medical Microbiology and Molecular Medicine, Department of Clinical and Experimental Medicine, Linköping University, Linköping, Sweden; ^2^Center for Infectious Medicine, Karolinska Institute, Stockholm, Sweden

**Keywords:** matrix metalloproteinases, tuberculosis, tissue inhibitor of matrix metalloproteinases, tissue models, granuloma

## Abstract

Granulomas are hallmarks of pulmonary tuberculosis (TB) and traditionally viewed as host-protective structures. However, recent evidence suggest that *Mycobacterium tuberculosis* (Mtb) uses its virulence factors to stimulate the formation of granuloma. In the present study, we investigated the contribution of matrix metalloproteinases (MMPs), host enzymes that cause degradation of the extracellular matrix, to granuloma formation and bacterial load in Mtb-infected tissue. To this end, we used our lung tissue model for TB, which is based on human lung-derived cells and primary human monocyte-derived macrophages. Global inhibition of MMPs in the Mtb-infected tissue model reduced both granuloma formation and bacterial load. The infection caused upregulation of a set of MMPs (MMP1, 3, 9, and 12), and this finding could be validated in lung biopsies from patients with non-cavitary TB. Data from this study indicate that MMP activation contributes to early TB granuloma formation, suggesting that host-directed, MMP-targeted intervention could be considered as adjunct therapy to TB treatment.

## Introduction

Immune responses in human tuberculosis (TB) are mainly studied in easily accessible peripheral blood samples, while the center of attention instead is staged in *Mycobacterium tuberculosis* (Mtb)-infected tissues where the granulomatous inflammation occurs. Granulomas are organized clusters of immune cells considered as pathological hallmarks at the site of Mtb infection, especially in human TB. Although it was initially anticipated that the function of the granuloma was solely to wall off the infection, it has become clear that early TB granuloma formation can instead favor dissemination of the bacteria in the tissue (Davis and Ramakrishnan, 2009). Consequently, late stage necrotic granulomas are a prerequisite for effective spread of Mtb in the local tissue environment (Pagan and Ramakrishnan, 2014). We and others have previously demonstrated that the ESX-1 gene-encoded ESAT-6 protein is essential for granuloma formation, while avirulent mycobacteria lack this protein and display a more moderate capacity to induce granuloma in infected tissue (Davis and Ramakrishnan, 2009; Parasa et al., 2014). Together, this reveals aspects of TB granuloma formation that favor the establishment of Mtb infection in tissues.

Matrix metalloproteinases (MMPs), a family of zinc-dependent proteases that are involved in tissue extracellular matrix destruction and remodeling (Elkington and Friedland, 2006; Volkman et al., 2010; Ong et al., 2014), have been implicated in pulmonary pathology of TB including granuloma formation (Elkington and Friedland, 2006; Volkman et al., 2010; Elkington et al., 2011). These proteases are regulated and inhibited by tissue inhibitors of MMPs (TIMPs) in order to maintain tissue homeostasis (Gaffney et al., 2015). An enhanced activity of MMPs has mainly been associated with extensive tissue damage in the lung leading to dissemination of TB (Elkington and Friedland, 2006; Ugarte-Gil et al., 2013). Elevated levels of MMPs have been reported in TB patients' serum (Patil et al., 2015), sputum (Walker et al., 2012; Ugarte-Gil et al., 2013), pleural fluid (Sundararajan et al., 2012), and bronchoalveolar lavage fluid (Singh et al., 2014). The levels of MMP1 in sputum samples from TB patients drop during anti-TB chemotherapy, consistent with the resolution of inflammation and pulmonary pathology (Ugarte-Gil et al., 2013). Several previously published studies correlated the activity of MMPs mainly to extensive lung tissue damage leading to dissemination of TB (Elkington and Friedland, 2006; Ugarte-Gil et al., 2013). We hypothesized that MMPs are required for early granuloma formation and to promote the propagation of Mtb bacilli in the local microenvironment. To explore the expression and effects of specific MMPs and TIMPs in Mtb-infected tissue, we used our recently developed experimental human lung tissue model for early TB granuloma formation (Parasa et al., 2014; Braian et al., 2015). This model is composed of epithelial and fibroblast cell lines, human monocytes, and differentiated macrophages, cultured in a bed of extracellular matrix components. We investigated the role of a panel of MMPs and TIMPs in Mtb-induced granuloma formation and if blocking the activity of specific MMPs could alter the process of granuloma formation and perturb Mtb growth. Results from this study suggest that MMP activity contribute to TB granuloma formation and that inhibitors of MMPs have the potential to reduce mycobacterial growth.

## Materials and methods

### Clinical samples

Monocytes were isolated from peripheral blood from healthy donors collected at Linköping University Hospital, after obtaining written consent for research use. Lung tissue biopsies (pathological lesion and distal non-pathological tissue) were obtained during curative surgery of patients (*n* = 19) with chronic pulmonary TB at the St. Petersburg State City TB Hospital in St. Petersburg, Russia, as previously described (Andersson et al., 2007; Rahman et al., 2015). A confirmed TB diagnosis in HIV-negative patients was based on clinical symptoms (chronic cough, general illness, fever, and weight loss), positive sputum-microscopy, Mtb culture, and/or Mtb-specific PCR as well as histopathology and chest X-ray findings consistent with TB. Symptoms persisted despite standard treatment with anti-TB drugs, which suggested MDR-TB and alternative surgical treatment to reduce disease burden (Andersson et al., 2007). Briefly, macroscopically normal areas in the peripheral regions of the resected lung (i.e., the same lung lobe) were defined as distal control parenchyma. This selection was based upon a visual examination performed by the clinicians to obtain a pathological biopsy from the granulomatous-fibrotic tissue areas and a non-pathological biopsy from distal lung parenchyma. Microscopy analysis confirmed that the TB lesions contained a necrotizing granulomatous inflammation characteristic of TB, while the distal tissues maintained the structure of uninflamed lung parenchyma. Patients were recruited after signed informed consent and ethical approval from the local ethical review boards in St. Petersburg and Stockholm.

### Cells

The MRC-5 human lung fibroblast cell line and the 16HBE human bronchial epithelial cell line (16HBE) were cultured and maintained as previously described (Parasa et al., 2014). The cells were seeded into the lung tissue model at passage number 20–24 and 78–80, respectively. Blood-derived monocytes were differentiated into macrophages as previously described (Welin et al., 2008). When fresh monocytes were required for addition into lung tissue model for investigating granuloma formation, blood-derived monocytes were enriched as previously described (Parasa et al., 2014).

### Mtb strains and infection of macrophages

The laboratory Mtb strain, H37Rv, carrying the green fluorescent protein (GFP)-encoding pFPV2 plasmid (Mtb-GFP) or the luciferase encoding pSMT1 plasmid (Mtb-lux) (Snewin et al., 1999; Eklund et al., 2010), were cultured in Middlebrook 7H9 medium supplemented with Tween-80, glycerol, albumin, dextrin, and catalase enrichment (BBL Middlebrook) as previously described (Eklund et al., 2010). For infection of human primary macrophages, mycobacteria were harvested and washed, resuspended in antibiotic-free complete DMEM and passed through a 27-gauge needle to break bacterial clumps. Macrophages were infected with Mtb at a concentration of MOI 10 for 4 h and the Mtb-infected macrophages were used for the lung tissue model experiments. Experiments with virulent Mtb were performed in a biosafety (BSL-3) facility available at Linkoping University in Sweden. For infection, the harvested bacteria were washed, resuspended in antibiotic-free complete DMEM and passed through a 27-gauge needle to break bacterial clumps. Macrophages were infected with Mtb at a concentration of MOI 10 for 4 h.

### Lung tissue model and MMP inhibition

The lung tissue models were prepared as previously described (Braian et al., 2015). In brief, 16HBE cells and human primary macrophages (uninfected/infected at MOI 10 with Mtb expressing GFP), PKH26 red dye-labeled monocytes (stained according to manufacturer's instructions) were seeded into a matrix of collagen-embedded MRC-5 cells. During tissue development, the models were air-exposed at day 5 post infection allowing mucus secretion and stratification (Nguyen Hoang et al., 2012; Parasa et al., 2014). At day 7 after infection, the models were harvested and fixed with 4% paraformaldehyde for 30 min. Supernatants were collected and stored at −80°C for cytokine and chemokine analysis. For MMP inhibition experiments, primary macrophages treated with a global MMP inhibitor, 200 nM marimastat (Merck Millipore), 16 h prior to Mtb infection were introduced in the lung tissue model. After the addition of macrophages, the tissue models were cultured in the presence of the inhibitor until tissue harvest.

### Assessment of granuloma formation using confocal microscopy

Upon harvest, the lung tissue models were cut into four pieces, transferred onto glass slides, stained with 4′,6-diamidino-2-phenylindole (DAPI from Invitrogen, Carlsbad, USA) and mounted. The samples were visualized using a Zeiss LSM700 Confocal system (Zeiss, Gottingen, Germany). Images were obtained at a 512 × 512 resolution with Z-stacks covering 20 μm thickness of tissue at 1.5 μm separation between the consecutive stacks. Images from six to nine microscopic fields were acquired from each tissue. The images of uninfected and infected tissues were analyzed using Imaris Image processing software Version 8.0 (Bitplane AG, Switzerland). 3D analysis of the size of red monocyte clusters reflecting the distribution of cells was done as described below. We used two approaches to quantify clustering of PKH26-positive cells. In the first approach (Braian et al., 2015), which allows quantification of clustering irrespective of infection, the volume of the cells/clusters is determined by creating Z-stacks of the PKH26 stain using confocal microscopy. The fields were visualized in the microscope using the blue channel followed by the green channel focusing on cells as well as bacteria and the acquired Z-stacks were analyzed in the red channel to determine the volume of monocytes/macrophages labeled with the red-fluorescent dye. To avoid possible bias, we used an alternative approach to quantify clustering by determining the mean fluorescent intensity (MFI) in Z-stacks obtained in the red channel in manually selected regions of interest (ROI) with bacteria (MFIbact) and the MFI in an adjacent ROI without bacteria (MFIcon) in the same microscopy field (internal controls). In this approach, which is a slight modification of our previously established method (Parasa et al., 2014), images were analyzed using the NIS Elements image analysis software (Nikon). To ensure unbiased analysis, selections of the ROIs were made in the green channel (bacteria) and then the MFI within the ROI was measured in the red channel (PKH26). All the ROIs were from cellular regions of the tissue as identified in the blue channel (DAPI, cell nuclei). As the tissue volume used for the measurement of MFIbact and MFIcon was identical for individual measurement pairs (MFIbac and MFIcon), ratios were calculated to compare the recruitment of monocytes/macrophages in different experiments.

### Immunohistochemistry of MMPs in the experimental model and patient tissues

For immunohistochemistry, lung tissue models and patient tissues embedded in OCT compound, snap frozen in liquid nitrogen and stored at −80°C were used. Staining of 8 μm thick cryosections were performed after fixation with 4% formaldehyde (Sigma, Stockholm, Sweden) as previously described (Andersson et al., 2007; Rahman et al., 2009, 2015). Staining of the cryosections were performed according to a modified protocol from a previous study (Parasa et al., 2014). Primary antibodies used were mouse monoclonal anti-human MMP1, 3, and 12 (25 μg/ml; R&D systems, Abingdon, UK), overnight incubation or rabbit polyclonal anti-MMP9 (25 μg/ml; Abcam), 2 h incubation. CD3- and anti-Mtb staining was done as previously described (Rahman et al., 2015). Tissue sections without primary antibodies were included as controls. Detection was done using goat anti-mouse or anti-rabbit secondary antibodies conjugated to horse radish peroxidase (Dako, Glostrup, Denmark) and peroxidase activity was visualized using 3,3′ diamino benzidine and H_2_O_2_ (brown precipitates). Bright-field images were obtained using a Leica DMR-X microscope (Leica Microsystems GmbH, Wetzlar, Germany) and analyzed by acquired computerized image analysis (Leica Qwin 5501W, Leica Microsystems GmbH, Wetzlar, Germany) as previously reported (Andersson et al., 2007; Rahman et al., 2009, 2015). This quantitative method allows an estimation of stained positive cell area out of the total tissue area.

### Screening of MMPs by real time PCR

Mtb-infected and uninfected tissue models were cut into small pieces of around 2 mm and then transferred to a sterile tube containing 1.5 mg/ml collagenase A (Roche, Basel, Switzerland) and DNase I (Thermofisher, Carlsbad, USA) in antibiotic-free DMEM as previously described in antibiotic-free DMEM (Nguyen Hoang et al., 2014). To facilitate digestion, the tubes were placed at 37°C for 30 min, constantly shaking at 150 rpm. After incubation, the enzymes were inactivated by the addition of 5 mM EDTA. The cell suspension was passed through 100 μm sterile filters and washed again with 2 mM EDTA. Six replicates of tissue models were pooled from Mtb-infected and uninfected samples respectively, to have sufficient material for the MMP/TIMP screening. To isolate individual cell types, sequential magnetic separation (Dynabeads, Carlsbad, USA) of monocyte/macrophages (CD14 beads) and epithelial cells (EpCam) was performed after incubation with respective antibodies (Rahman et al., 2015), leaving behind the unbound fibroblasts. The purified fractions were lysed with TRIzol (Life Technologies, Carlsbad, USA), total RNA extracted (ZymoResearch), reverse transcribed to cDNA and the mRNA expression of MMP1, 2, 3, 7, 8, 9, 12, 13, and 14 and TIMP1, 2, and 3 (Taqman gene expression probes from Applied Biosystems, Carlsbad, USA) were determined using real-time PCR as described earlier (Nguyen Hoang et al., 2012). For comparison, cryopreserved clinical biopsies were sectioned, RNA extracted, reverse transcribed, and the gene expression of the above MMPs and TIMPs were determined.

### Bacterial load

Bacterial replication in the tissue models and the effect of marimastat was assessed using a modification of the previously established luminescence-based method (Eklund et al., 2010). Luciferase-expressing Mtb (Mtb-lux) were used to infect tissue models in the presence or absence of marimastat. After Mtb-infection and marimastat exposure (16 h prior to infection of macrophages and thereafter until tissue harvest), the tissues were digested as described above. Luminescence emitted by live bacteria in the supernatants (obtained after pelleting the cells after tissue digestion) and cell lysates (obtained through addition of sterile water to the cells for 10 min) was measured using a plate reader (GloMax-Multi + Detection System with Instinct Software, Promega, Stockholm, Sweden) after injection of the luciferase substrate, decanal (1%). Measurements were performed at day 1 and 7 post infection to determine the fold change of bacterial growth. In validation experiments, a portion of the supernatants and lysates were also plated on Middlebrook 7H10 agar plates to determine bacterial numbers by assessing colony forming units.

### Detection of cytokines and chemokines

Cytometric bead array kits (BD, San Diego, USA) were used as per manufacturer‘s instructions to detect cytokines and chemokines in lung tissue model supernatants. The cytokines (IL-1β, IL-6, IL-12, and TNFα with minimum detection limit of 2.3, 1.6, 7.9, and 0.7 pg/ml, respectively) and chemokines (RANTES, IL-8, IP-10 and MCP-1 with minimum detection range of 0.002, 1.2, 0.5, and 1.3 pg/ml, respectively) were analyzed using a Gallios flow cytometer and the Kaluza analysis software (Beckman Coulter, Stockholm, Sweden).

### Statistical analysis

Statistical analysis was performed with the GraphPad Prism software version 6.0 (GraphPad, San Diego, CA). Two-way ANOVA with Sidak's multiple comparisons test was used to compare within and between groups and one-way ANOVA with Tukey's multiple comparisons test was used to for comparisons between groups. Wilcoxon's signed rank test was used for statistical testing in experiments where we present ratios or fold change. Student's *t*-test for paired samples was used when comparing the stained area of biopsies. Data is presented as mean or median of individual groups and is indicated in the figure legends along with the statistical test used. The threshold for significant *p*-value is as follows ^*^p ≤ 0.05, ^**^p ≤ 0.01, ^***^p ≤ 0.001, ^****^p ≤ 0.0001.

## Results

### Blocking of MMPs inhibits monocyte clustering and Mtb replication in the tissue model

**Figure 1:**
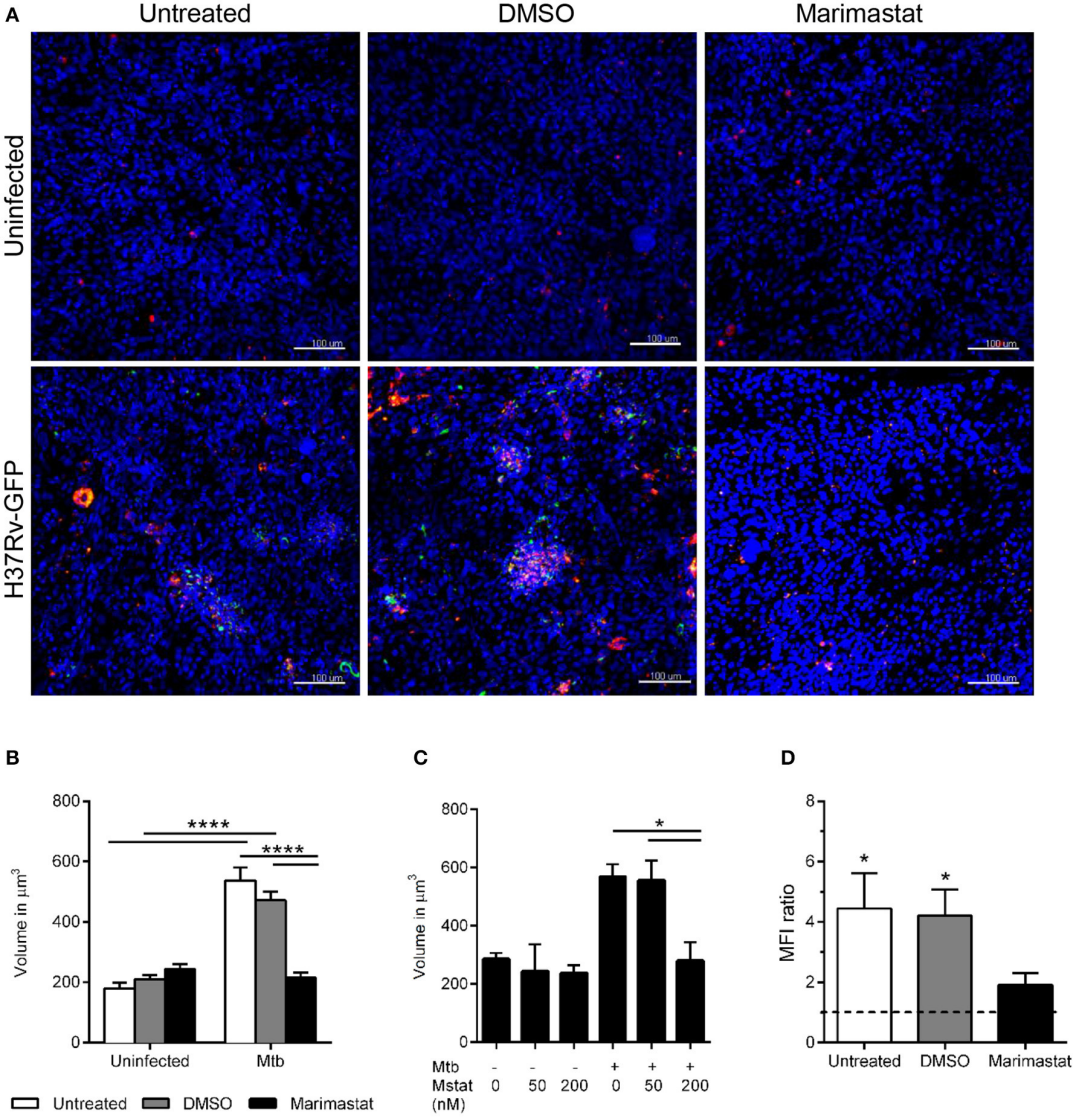
Effect of marimastat on early granuloma formation in Mtb-infected lung tissue models. **(A)** Tissue models (nuclei stained with DAPI, blue) were left untreated or incubated with DMSO or marimastat before implantation with PKH26-labeled monocytes (red) and macrophages, uninfected or infected with GFP-expressing (green) H37Rv. The tissues were fixed at day 7 and subjected to DAPI-staining followed by confocal microscopy. Images show merged confocal images of randomly selected fields (uninfected models) or bacteria-harboring fields selected in the green channel (H37Rv-GFP-infected models) with treatments as indicated. **(B)** Z-stacks were obtained in the red channel and the volume of the cells/clusters was determined in uninfected and infected samples with or without 200 nM marimastat (*n* = 13). **(C)** Two different concentrations of marimastat (Mstat), 50 and 200 nM were tested (*n* = 3). **(D)** Regions of interest (ROIs) with (ROI_bact_) and without bacteria (ROI_con_) were selected in the green channel and the MFI in the red channel was determined. Recruitment of monocytes/macrophages to the site of infection is expressed as ratio of MFI (ROI_bact_)/MFI (ROI_con_, *n* = 6). Data are expressed as means + SEM. Statistical analyses were carried out using 2-way ANOVA with Sidak's multiple comparisons test for comparisons within and between groups **(B)**, 1-way ANOVA with Tukey's multiple comparisons test within the infected group **(C)** and Wilcoxon signed rank test to compare MFI ratio **(D)**. Scale bars are 100 μm. ^*^p ≤ 0.05, ^****^p ≤ 0.0001.

To assess whether MMPs contribute to TB granuloma formation, developing tissue models were left untreated or incubated with DMSO (vehicle control) or 200 nM marimastat as indicated in Figure 1. PKH26-labeled (red) monocytes were added along with uninfected or Mtb-infected macrophages and the models were incubated 7 days. Confocal analysis was performed after fixation of the tissues to assess aggregation of the PKH26-labeled monocytes/macrophages in the models (Figure 1A and Supplementary movie 1). In order to quantify clustering of these cells we use two approaches. In the first approach, the size of the red cells/clusters is determined by measuring the volume in Z-stacks obtained by confocal microscopy. This allows assessment of cell clustering in both non-infected and infected tissues. Using this approach, we confirmed our observations from previous studies (Parasa et al., 2014; Braian et al., 2015) that Mtb induces clustering of monocytes/macrophages at the site of infection (Figures 1A,B). Marimastat treatment, however, caused a significant reduction in cluster size in the infected models (Figures 1A,B, *p* < 0.05), indicating that inhibition of MMPs prevents early granuloma formation. A lower concentration of marimastat (50 nM) did not have any effect (Figure 1C).

An alternative approach to quantify clustering is to determine the MFI in Z-stacks obtained in the red channel in manually selected ROI with bacteria (MFI_bact_) and the MFI in an adjacent ROI without bacteria (MFI_con_) in the same microscopy field (internal controls). This approach better ensures unbiased analysis and allows assessment of the relative distribution of cells in infected/non-infected areas. Thus, the ROIs were selected in the green channel (GFP-Mtb) and then the Z-stack MFI (cells) was measured in the red channel in the same ROI. The calculated MFI ratio was significantly elevated in Mtb-infected tissue models without pharmacological treatment (no treatment/DMSO), whereas this effect was not observed during inhibition of MMPs using marimastat (Figure 1D).

**Figure 2:**
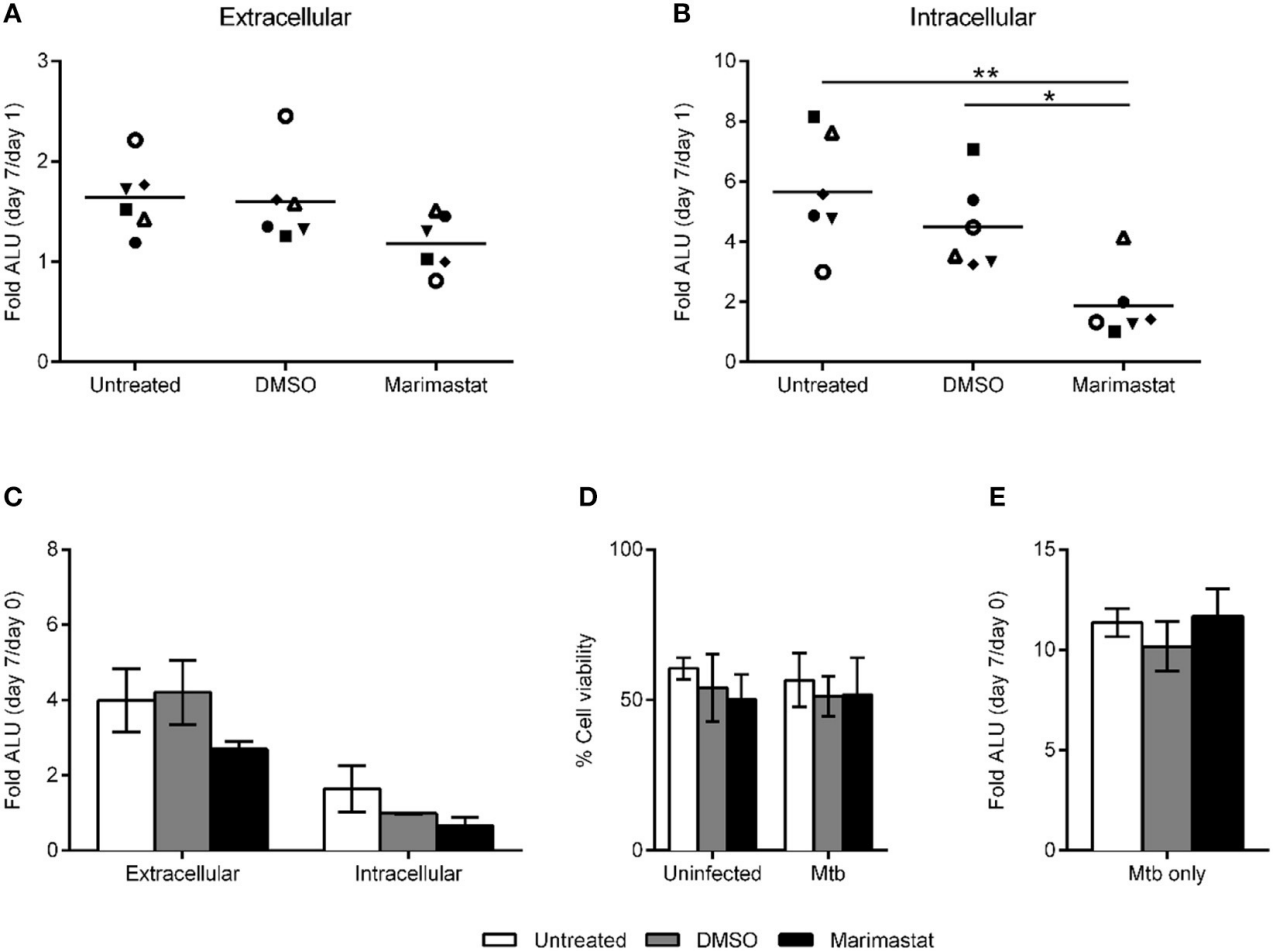
Effect of MMP inhibition on bacterial growth. Tissue models were left untreated, mock-treated (DMSO) or treated with 200 nM marimastat, infected with Mtb-lux, digested and analyzed with respect to bacterial replication after 1 and 7 days post infection. The fold change in bacterial numbers expressed as the ratio of arbitrary luminescence units (ALU) from day 7/Day 1 (*n* = 6) was determined in the extracellular matrix **(A)** and intracellularly **(B)**. **(C)** Macrophage monocultures were treated as indicated and infected with Mtb-lux. ALU was determined in supernatants (extracellular) and in cell lysates (intracellular) at day 0 and 7 and the fold change in bacterial numbers was determined by calculating the ratios of ALU at day 7/day 0 (*n* = 3). **(D)** The viability of these macrophages was determined by measuring the uptake of calcein-AM. **(E)** Broth cultures of Mtb-lux were left untreated or incubated with DMSO or marimastat and the fold change in bacterial numbers expressed as the ratio of arbitrary luminescence units (ALU) from day 7/day 0. Statistical analysis was done using 1-way ANOVA with Tukey's multiple comparisons test between groups (**A,B**,**E**, ^*^p ≤ 0.05, ^**^p ≤ 0.01) and 2-way ANOVA with Sidak's multiple comparisons test within and between groups (**C,D**, error bars are SEM).

We next analyzed whether inhibition of granuloma formation by marimastat affects Mtb growth in the tissues. To address this, we digested marimastat- or mock-treated tissue models infected with Mtb-lux (MOI 10) at day 1 and 7 post infection. Bacterial growth was determined by luminescence measurement in supernatants (reflecting growth in the extracellular matrix) and in lysates of the collected cells (reflecting intracellular growth). As shown in Figure 2A, marimastat did not significantly affect the extracellular population of bacteria (supernatant), however we could observe a significant reduction in intracellular bacterial load (lysate) as compared to untreated or DMSO controls (Figure 2B). The result was confirmed using CFU plating, showing that significant Mtb growth between day 1 and 7 took place in untreated and DMSO-treated tissue models, but not in marimastat-treated models. In control experiments, we analyzed the effect of marimastat on Mtb-lux replication inside macrophages. No significant difference could be observed between the mock- or marimastat-treated infected macrophages (Figure 2C). Neither treatment (DMSO or 200 nM marimastat) affected cell viability as determined by calcein-AM uptake (Figure 2D). We also analyzed the direct effect of marimastat on the growth of Mtb-lux by incubating bacterial broth cultures with or without DMSO or 200 nM marimastat for 7 days, measuring the luminescence at day 0 and 7. However, there was no significant difference in the D7/D0 ratios between the different conditions (Figure 2E).

### Cytokine and chemokine levels are not altered by global MMP inhibition

**Figure 3:**
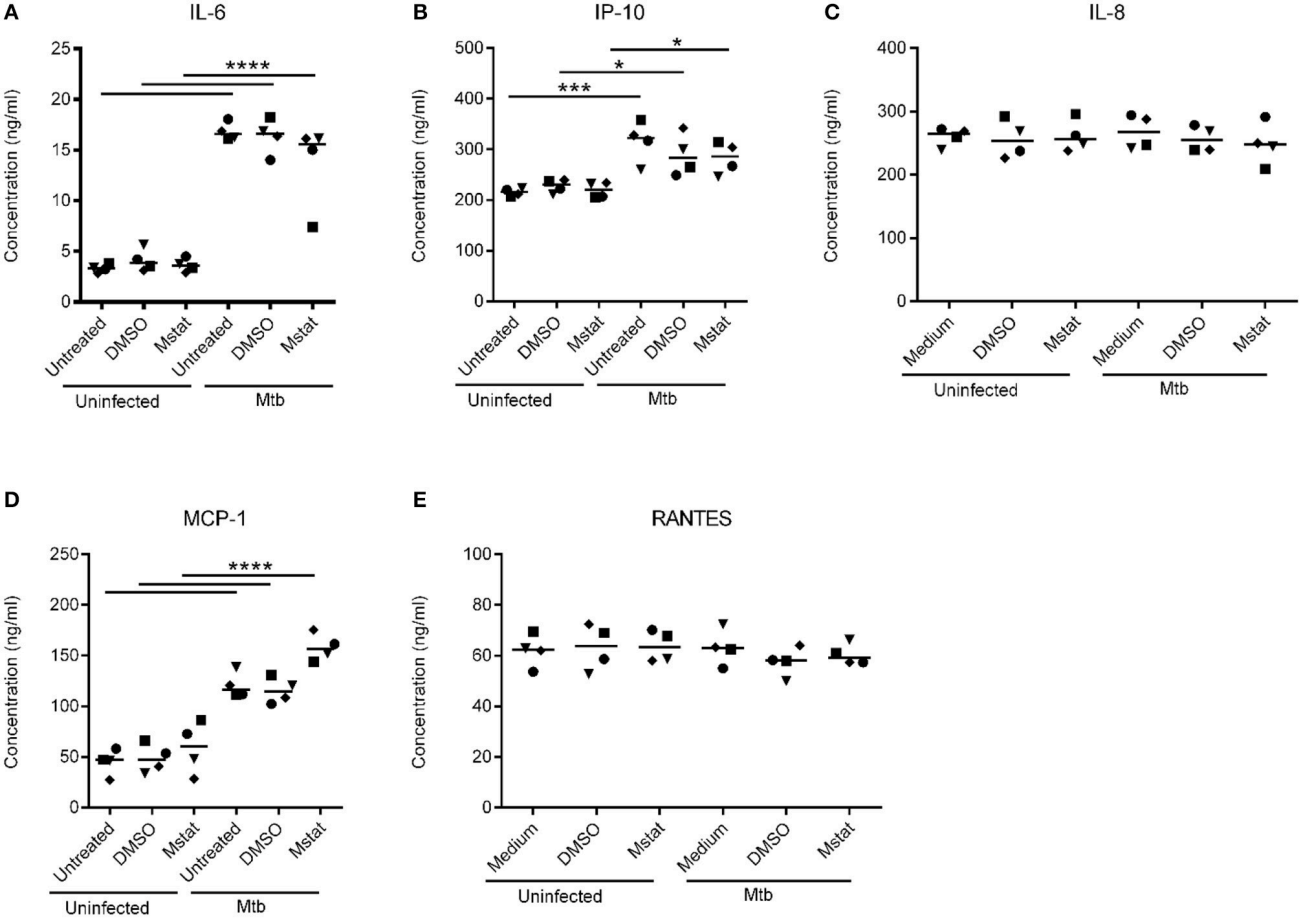
Cytokine and chemokine levels in Mtb-infected lung tissue models. Cytokine Bead Array analyses were performed to measure cytokine and chemokine levels in the medium supernatant of lung tissue models (both Mtb-infected and uninfected) that were treated with 200 nM marimastat (Mstat) or mock (untreated or DMSO). The concentrations of IL-6 **(A)**, IP-10 **(B)**, IL-8 **(C)**, MCP-1 **(D)**, and RANTES **(E)** are shown as means (lines) and individual symbols from four experiments. Statistical analyses were done using 2-way ANOVA with Sidak's multiple comparisons test. ^*^p ≤ 0.05, ^***^p ≤ 0.001, ^****^p ≤ 0.0001.

Next, we assessed the levels of cytokines and chemokines relevant to TB in the lung tissue model supernatants of infected and non-infected models with or without treatments. The level of IL-6 was found to be significantly elevated in Mtb-infected tissue models compared to uninfected controls, however marimastat treatment did not block the effect (Figure 3A). Besides IL-6, other pro-inflammatory cytokines, IL-1β, TNF-α, IL-12p40, were measured but the levels were below detection limit in all samples (not shown). Four chemokines representing both the CXC (IP-10 and IL-8) and C (MCP-1 and RANTES) families were analyzed. IP-10 and MCP-1 were significantly increased in response to the infection (Figures 3B,D). However, like observed with IL-6, marimastat did not inhibit the effect. IL-8 and RANTES were not altered in response to infection or treatment (Figures 3C,E).

### Expression of MMPs and TIMPs in Mtb-infected lung tissue models

**Figure 4:**
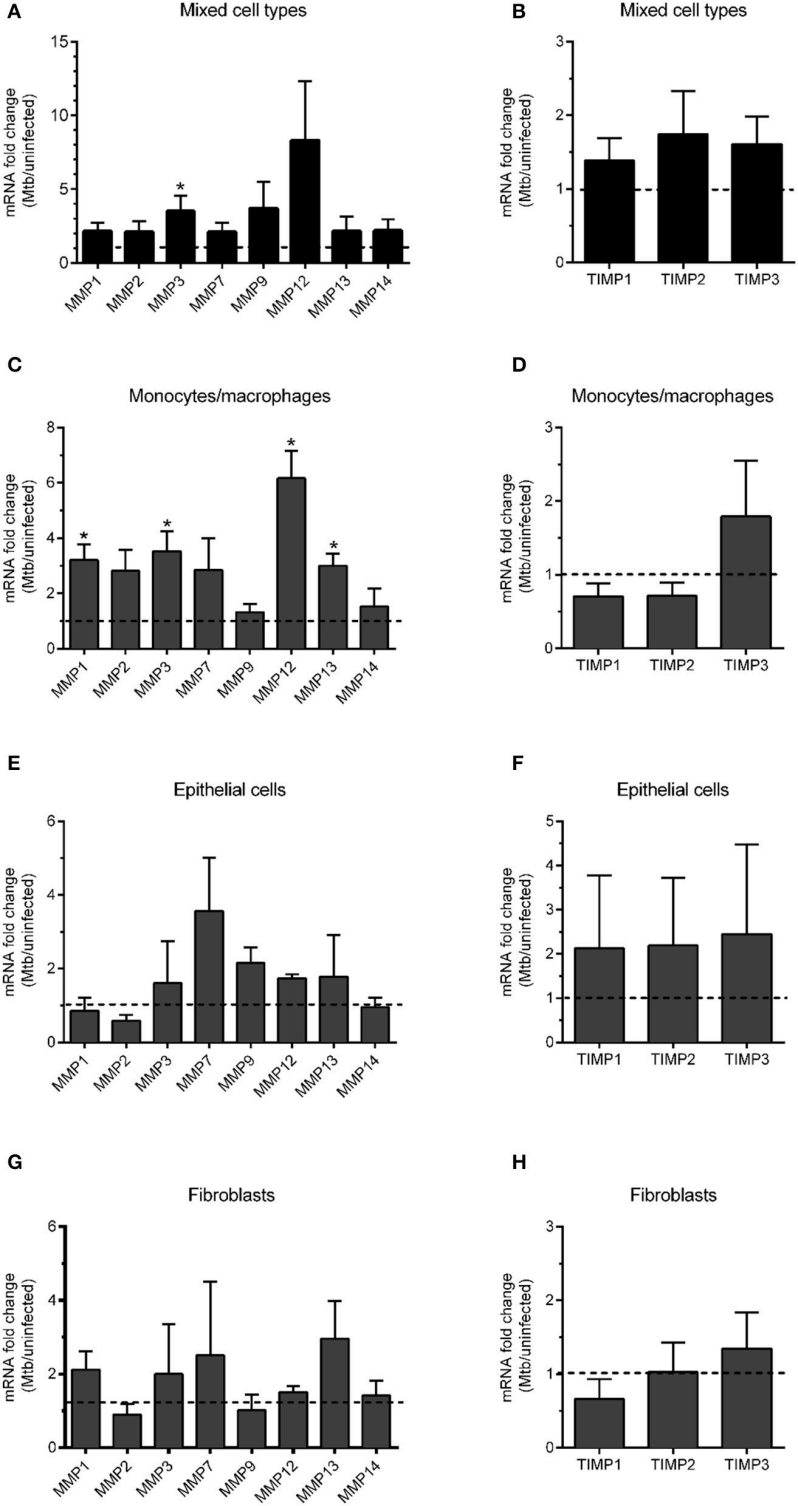
mRNA transcript analysis of MMPs and TIMPs in Mtb-infected lung tissue models. mRNA expression of a panel of MMPs and TIMPs in Mtb-infected tissue models was analyzed (*n* = 3). mRNA was extracted either from the whole tissue model (“Mixed cell types”, **A,B**) or from individual cell types isolated from digested tissue (**C,D** macrophages; **E,F** epithelial cells and **G,H** fibroblasts). Data are presented as mean + SEM of fold change in mRNA expression normalized to uninfected tissue models (dotted line) (*n* = 6). Statistical analysis was done using Wilcoxon-Signed Rank test. ^*^p ≤ 0.05.

In order to analyze which MMPs are upregulated during Mtb infection in the lung tissue model, we digested the tissue models that were either uninfected or infected with Mtb and analyzed the mRNA expression of a selection of MMPs and TIMPs relevant for the cell types included in the model. The selection included four collagenases (MMP1, 8, 13, and 14), two gelatinases (MMP2, 9) and stromelysin (MMP3), matrilysin (MMP7) and metalloelastase (MMP12), and three TIMPs (TIMP1, 2, and 3). mRNA analysis performed using lysates of the mixed cell types from the digested tissue revealed that MMP3 was significantly elevated in infected tissues. The levels of MMP9 and MMP12 showed a trend toward increase (Figure 4A). The same analysis was performed for the selected TIMPs, however, no significant difference could be observed between infected and non-infected tissues (Figure 4B). To get a better resolution of the data and better understand the source of the MMP release, we instead isolated the different cell types included in the model (monocytes/macrophages, epithelial cells and fibroblasts) and lysed and analyzed the mRNA content of the individual cell types. The analysis showed that MMP1, 3, 12, and 13 were clearly (>3-fold) and significantly upregulated in macrophages isolated from infected tissues (Figure 4C), but none of the tested TIMPs (Figure 4D). The TIMPs were not upregulated in epithelial cells (Figure 4F) or fibroblast (Figure 4H) either. MMP7 and MMP13 were 3-fold upregulated in epithelial cells (Figure 4E) and fibroblasts (Figure 4G), respectively, however, the analysis did not reach statistical significance.

**Figure 5:**
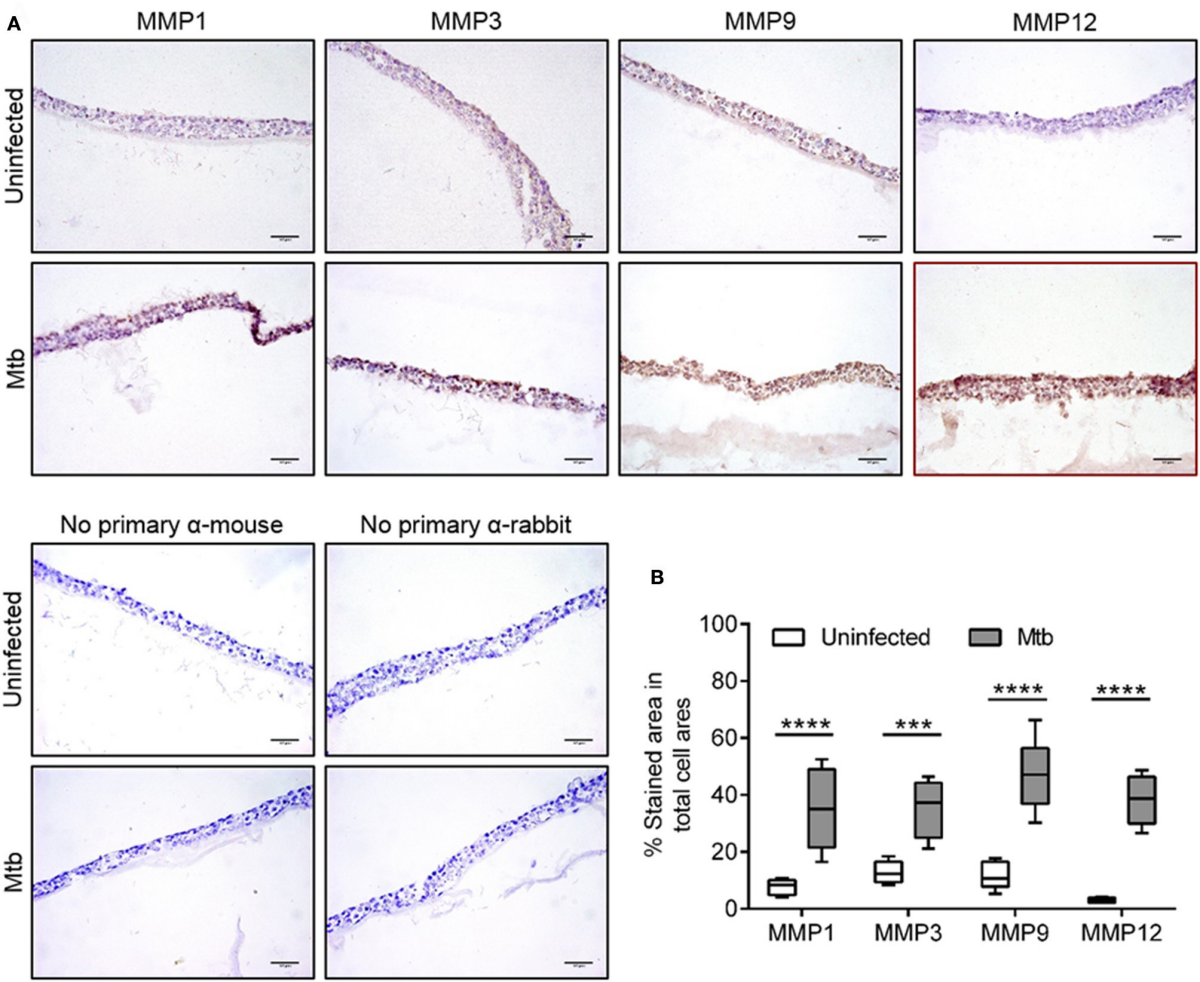
Immunohistochemistry and *in-situ* quantification of MMPs in lung tissue models. **(A)** Representative images of cryosectioned lung tissue models stained for MMP1, 3, 9, and 12 using immunohistochemistry (MMP-positive areas in brown color and hematoxylin-stained cellular areas in blue color). **(B)** Quantitative analysis of MMP-positive areas was performed by measuring the percentage of brown colored-area/total blue-colored area. Data are collected from eight fields per tissue section and are presented as median of 6 experiments (lines) with interquartile range (boxes). Statistical comparison of MMP-positive area in uninfected and Mtb-infected tissue models was performed using 2-way ANOVA with Sidak's multiple comparison test. Scale bar is 50 μm. ^***^p ≤ 0.001, ^****^p ≤ 0.0001.

In order to test whether the observed mRNA alterations could be matched with increased upregulation at the protein level, we performed immunohistochemical analysis of the infected and non-infected tissue models using a selection of relevant MMPs for which reliable antibodies are available (MMP1, 3, 9, and 12). While MMP1, 3, and 9 showed a relatively higher baseline expression compared to MMP12 in uninfected models, all tested MMPs showed an increased expression in Mtb-infected compared to uninfected tissue models (Figure 5A). Control experiments were performed in which the primary antibody was omitted. Quantification of the area of positive staining was performed in the images obtained from the tissue models (Figure 5B). The analysis showed that all tested MMPs were significantly upregulated in Mtb-infected tissue models. Hence, for MMP1, 3, and 12, the data is conclusive for both mRNA and protein expression.

### Tissues from TB patients express MMPs and TIMPs

**Figure 6:**
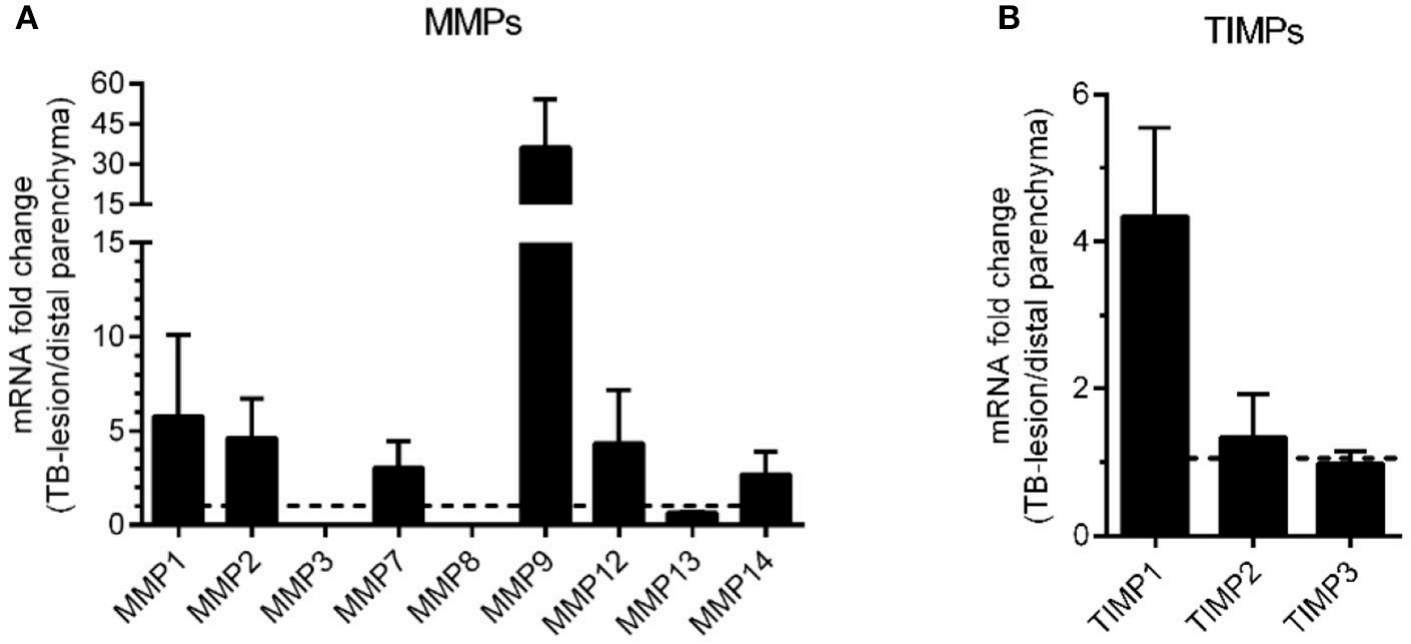
mRNA transcript analysis of biopsies from TB patients. Granulomatous TB lesion lung biopsies from infiltrative non-cavitary TB patients (*n* = 4) were analyzed for mRNA expression of MMPs **(A)** and TIMPs **(B)**. The mRNA levels were normalized to data from lesion-free distal lung parenchyma of the same patients (dotted line) and are expressed as means with SEM.

**Figure 7:**
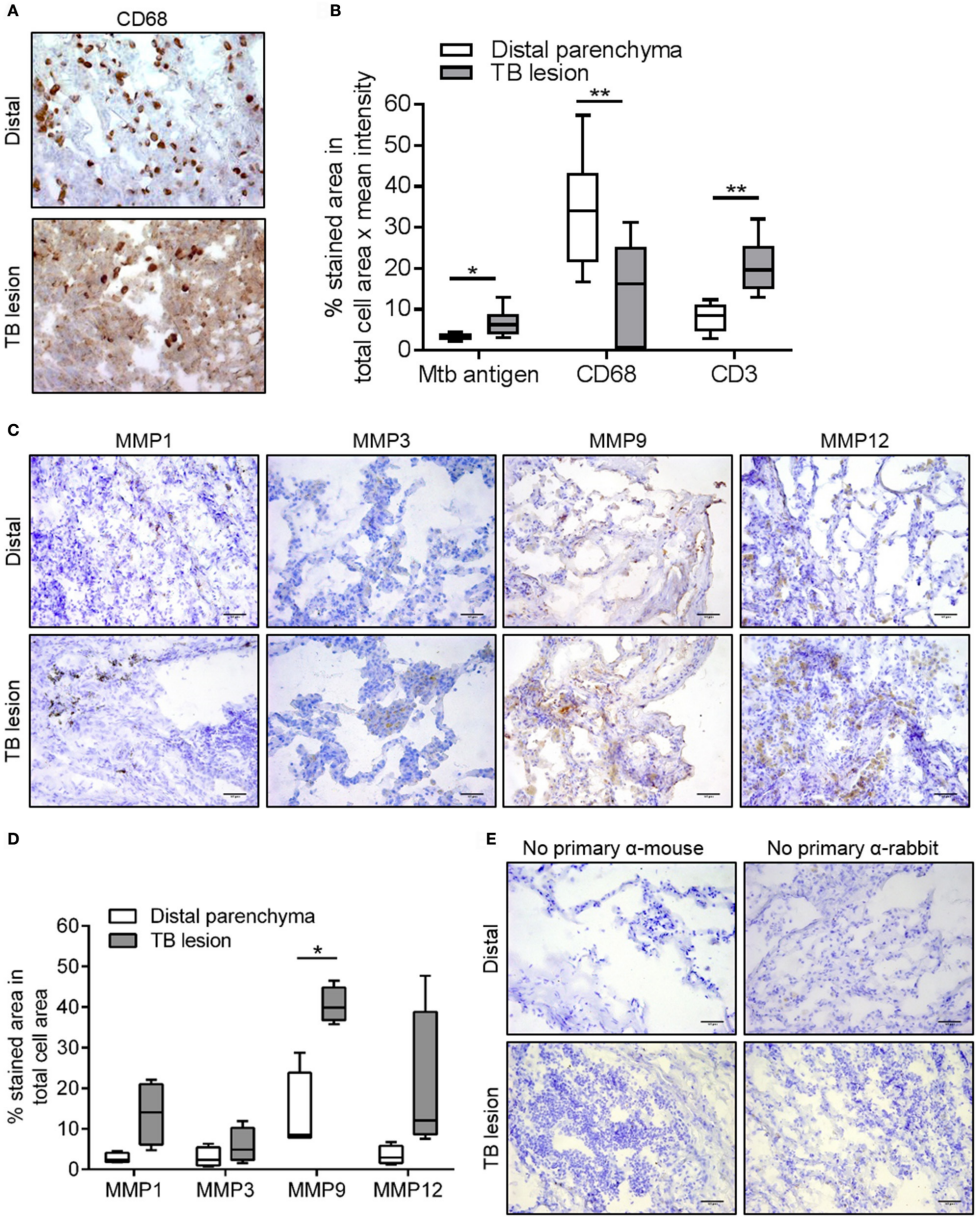
Immunohistochemistry of MMP1, 3, 9, and 12 in lung biopsies from TB patients. **(A)** Representative images of cryosectioned TB lesions and distal areas of lung tissue biopsies obtained from patients with non-cavitary pulmonary TB and stained with anti-CD68 antibodies using immunohistochemistry (CD68-positive areas are in brown color and hematoxylin-stained areas are in blue color). **(B)** Quantitative analysis of the cryosections of the biopsies using a BCG-specific antibody with cross-reactivity to Mtb (Mtb antigen), anti-CD68 (macrophages) and anti-CD3 (T cells). **(C)** Representative images of the cryosectioned TB lesions and distal areas of lung tissue biopsies stained with anti-MMP1, 3, 9, and-12. **(D)** Quantitative analysis of the MMP-positive areas expressed as percentage of brown colored-area/total blue-colored area. Data was obtained from 4 patients (10 fields/tissue section) and are shown as median (lines) with interquartile range (boxes). **(E)** In a control experiment, cryosections of the biopsies were stained without using the primary antibodies. Statistical comparison of TB lesions and distal parenchyma was performed using Student's *t*-test for paired samples. Scale bar–50 μm. ^*^p ≤ 0.05, ^**^p ≤ 0.01.

In order to evaluate whether our findings from the lung tissue model corresponds to human pulmonary TB, we moved on to analyze the expression of MMPs and TIMPs in matched lung tissue biopsies from TB lesion and distal sites of Mtb-infected lungs from patients with non-cavitary TB. Including the same MMPs and TIMPs as in the lung tissue model analyses above but adding MMP8 (a neutrophil-derived MMP that is potentially present in human TB lesions), we analyzed the mRNA expression patterns in TB lesions and distal regions of the lung biopsies. We found that MMP9 was strongly upregulated (more than 30-fold), whereas MMP1, 2, 7, and 12 displayed a more moderate upregulation (>3-fold upregulated in TB-lesions, Figure 6A). We also tested the mRNA expression of the relevant TIMPs, and found that TIMP1 was more than 4-fold upregulated in the lesions, whereas TIMP2 and 3 mRNA remained unchanged (Figure 6B). To better characterize the TB lesions and distal regions in the lung tissue biopsies, we investigated the presence of Mtb antigen and the expression pattern of CD68 and CD3 in cryosections from these regions (Figures 7A,B), showing an increased presence of Mtb antigens and CD3-positive cells and a reduction of CD68-positive cells in TB lesions, which is in line with previous observations (Andersson et al., 2007; Rahman et al., 2015). Finally, we addressed the protein expression levels of MMP1, 3, 9, and 12 using immunohistochemistry in the distal regions and TB-lesions of the lung biopsies (Figure 7C). Quantification of stained areas revealed that MMP9 was robustly and significantly upregulated, whereas MMP1, 3, and 12 were moderately but non-significantly upregulated in the TB lesions (Figure 7D), despite a reduced expression of CD68-positive macrophages in the lesions compared to distal sites. In control experiments, we assessed the background of the secondary antibody (Figure 7E). An overview of the obtained results on MMP upregulation in both the tissue model and biopsies is given in Table 1.

**Table 1:** The different samples analyzed in the study are listed.

**Sample/MMP**	**Analysis**	**1**	**2**	**3**	**7**	**8**	**9**	**12**	**13**	**14**
Mixed cells (whole tissue model)	mRNA	–	–	✓	–	nd	(✓)	(✓)	–	–
Monocytes/macrophages	″	✓	–	✓	–	nd	–	✓	✓	–
Epithelial cells	″	–	–	–	(✓)	nd	–	–	–	–
Fibroblasts	″	–	–	–	–	nd	–	–	(✓)	–
Whole tissue model	IHC	✓	nd	✓	nd	nd	✓	✓	nd	nd
Lung biopsies	mRNA	(✓)	(✓)	–	(✓)	–	(✓)	(✓)	–	–
″	IHC	(✓)	nd	(✓)	nd	nd	✓	(✓)	nd	nd

*Significant upregulation of individual MMPs are indicated as ✓, more than 3-fold, but not statistically validated upregulation is indicated as (✓). nd, No data, –, not upregulated*.

## Discussion

Granulomas in tissues are a characteristic feature of human TB, while cavitation of lung and extensive tissue damage are pathological features of advanced TB. Proteases, including MMPs, have been implicated in cavitary TB, with elevated levels found in sputum (Walker et al., 2012; Ugarte-Gil et al., 2013), broncho-alveolar lavage (Singh et al., 2014), pleural fluid (Sundararajan et al., 2012), and serum (Patil et al., 2015). These proteases are considered to be responsible for the destruction of tissue and extracellular structural proteins. The process of granuloma formation, an orderly recruitment of uninfected cells to an infection foci, also involves tissue remodeling most likely through the digestion of extracellular matrix. Granuloma formation has historically been considered as host protective, however, recent evidence suggest that Mtb uses secreted virulence factors to induce granuloma formation to promote its dissemination and replication (Davis and Ramakrishnan, 2009; Parasa et al., 2014). Thus, granuloma may provide a favorable niche for mycobacterial replication (Cronan et al., 2016). MMP1 has specifically been show to contribute to TB pathology in human lungs (Elkington et al., 2005, 2011). In this study, we elucidated the expression pattern of a panel of MMPs and TIMPs in an *in vitro* lung tissue model and in lung tissue biopsies from TB patients and evaluated the consequences of MMP inhibition for granuloma formation and bacterial load in the tissue model.

Marimastat (BB-2516) is a collagen-peptidomimetic drug that binds to the active site zinc atom of several MMPs, preventing protease activity. The anti-neoplastic properties of the drug has been extensively studied in clinical trials (Ramnath and Creaven, 2004), however it has not yet been approved for clinical use partly due to musculoskeletal side effects (Ong et al., 2014). In the present study, marimastat was selected due to its selectivity for MMPs alongside with its lack of specificity for the individual MMPs to reach a global inhibition of MMPs in the experiments. We show that marimastat at 200 nM blocked both granuloma formation and mycobacterial growth in our human lung tissue model for TB granuloma formation. Our observation is in line with a previous study based on the zebra fish model for mycobacterial granuloma, in which the authors showed that blocking of MMP activity inhibits the bacterial expansion by halting the macrophage recruitment and maturation of nascent granulomas (Volkman et al., 2010). Our finding is also coherent with a study in mice, in which MMP inhibition is shown to reduce granuloma formation (Hernandez-Pando et al., 2000).

Until date, no studies on TB have screened for MMPs in tissues at the cellular level. Here, we dissected the individual cell types and found the monocytes/macrophages to be the major source of MMPs. Of the panel of analyzed MMPs, a consistent pattern emerged from the Mtb-infected tissue model showing upregulation of MMP1, 3, 9, and 12. We were able to validate this finding in human lung tissue biopsies from patients with non-cavitary TB. The involvement of these MMPs in granulomatous tissues had been shown separately in different models including mice (Ramos-Martinez et al., 2015; Ordonez et al., 2016), rabbits (Kubler et al., 2015, 2016), fish (van der Sar et al., 2009; Volkman et al., 2010), and human tissues (Ong et al., 2015; Sathyamoorthy et al., 2015). Similar to our results, it has been shown that in human lung tissue biopsies of patients with TB, MMPs can be located in the macrophages surrounding the areas with caseous necrosis, (Elkington et al., 2005), at sites of tissue destruction (Green et al., 2010) and in cerebrospinal fluid during TB meningitis (Majeed et al., 2016). MMP9 is induced in epithelial cells surrounding growing granuloma, but its activity is dependent on the presence of macrophages (Elkington et al., 2007; Volkman et al., 2010). Although previously suggested (Ong et al., 2015), the neutrophil-derived MMP8 was not upregulated in the biopsies included in this study, however, the former work focused on cavitary TB, suggesting that the neutrophil-derived MMP can be linked to more advanced disease. Among the analyzed TIMPs, none was found to be upregulated in the Mtb-infected tissue model and TIMP1, but not TIMP2 or 3, was upregulated in lesions of non-cavitary TB patients. The role of TIMPs in TB remain obscure, however, Mtb has been suggested to actively dysregulate the balance between MMPs and TIMPs (Kubler et al., 2015). We did not find any significant changes in cytokine and chemokine secretion after inhibition with marimastat in the *in vitro* lung tissue model. However, more molecules have to be included to confirm this. Consistent to our data, a study comparing human MMP1-expressing mice and wild-type mice found no difference in cytokine/chemokine secretion (Al Shammari et al., 2015).

A limitation of the present study is that only a global MMP inhibitor was tested. Hence, the relative contribution of the activity of individual MMPs to granuloma formation and promotion of bacterial replication could not be dissected.

Of note, the MMPs consistently upregulated during Mtb infection in the present study cover four classes of MMPs, collagenase (MMP1), stromelysin (MMP3, a multi-substrate enzyme degrading several components of the extracellular matrix and also cleaving and activating several MMPs), MMP9 (gelatinase), and MMP12 (elastase). Interestingly, the same set of MMPs genes carry polymorphisms predisposing for multiple sclerosis (Mirowska-Guzel et al., 2009). Although the connection is speculative, we have previously found genetic polymorphisms that can be linked to inflammation that on the same time affect the outcome of Mtb infection (Eklund et al., 2014). TB, being a major cause of death among young adults in Europe a century ago, must have posed a substantial selective pressure on the population for individuals with genetic variants that limited the severity of the disease.

In summary, we conclude that marimastat inhibits granuloma formation and growth of Mtb in our tissue model for TB. Concomitantly, we show upregulation of MMP1, 3, 9, and 12 in the tissue model. The finding was validated in TB lesions of human non-cavitary TB. Further studies on the effects of MMP inhibitors on TB pathology and Mtb bacterial load are warranted to investigate the potential of MMP inhibition as an adjunct therapy that can be added to the standard TB regimen.

## Ethics statement

This study was carried out in accordance with the recommendations of the local ethics committees in Linkoping and Huddinge (Sweden) and St. Petersburg (Russia) with written informed consent from all subjects. All subjects gave written informed consent in accordance with the Declaration of Helsinki. The protocol was approved by the local ethics committees in Linköping and Huddinge (Sweden) and St. Petersburg (Russia).

## Author contributions

VP, SB, and ML conceived and designed the manuscript. VP, JM, and JR performed data acquisition. VP, JM, JR, and CB analyzed the data. VP, CB, SB, and ML interpreted the data. VP, JR, CB, SB, and ML wrote the manuscript. All authors approved the final version of the manuscript.

### Conflict of interest statement

The authors declare that the research was conducted in the absence of any commercial or financial relationships that could be construed as a potential conflict of interest.
