# Supplementary material for: Inhibition of Tissue Matrix Metalloproteinases Interferes with Mycobacterium tuberculosis-Induced Granuloma Formation and Reduces Bacterial Load in a Human Lung Tissue Model
Source: Front Microbiol. 2017 Dec 5;8:2370. doi: 10.3389/fmicb.2017.02370 (PMC5723394; doi:10.3389/fmicb.2017.02370)
Supplement: Supplementary file 1 [file Presentation1.pdf]

Movie caption

Movie showing rotating assemblies of Z-stacks obtained from confocal microscopy analysis of the lung tissue models: uninfected, Mtb-infected, Mtb-infected & mock-treated (DMSO), Mtb-infected & MMPI (marimastat)-treated. The infected models are stained with DAPI (nuclei), PKH27 (monocytes) and GFP (Mtb).
